# Taking a Radical Position: Evidence for Position-Specific Radical Representations in Chinese Character Recognition Using Masked Priming ERP

**DOI:** 10.3389/fpsyg.2012.00333

**Published:** 2012-09-18

**Authors:** I.-Fan Su, Sin-Ching Cassie Mak, Lai-Ying Milly Cheung, Sam-Po Law

**Affiliations:** ^1^Laboratory for Communication Sciences, Division of Speech and Hearing Sciences, The University of Hong KongHong Kong SAR, China

**Keywords:** word recognition, Chinese radicals, sub-lexical processing, orthography, spatial specification, N170, P200, N400

## Abstract

In the investigation of orthographic representation of Chinese characters, one question that has stimulated much research is whether radicals (character components) are specified for spatial position in a character (e.g., Ding et al., 2004; Tsang and Chen, 2009). Differing from previous work, component or radical position information in this study is conceived in terms of relative frequency across different positions of characters containing it. A lexical decision task in a masked priming paradigm focusing on radicals with preferred position of occurrence was conducted. A radical position that encompasses more characters than other positions was identified to be the preferred position of a particular radical. The prime that was exposed for 96 ms might share a radical with the target in the same or different positions. Moreover, the shared radical appeared either in its preferred or non-preferred position in the target. While response latencies only revealed the effect of graphical similarity, both effects of graphical similarity and radical position preference were found in the event-related potential (ERP) results. The former effect was reflected in greater positivity in occipital P1 and greater negativity in N400 for radicals in different positions in prime and target characters. The latter effect manifested as greater negativity in occipital N170 and greater positivity in frontal P200 in the same time window elicited by radicals in their non-preferred position. Equally interesting was the reversal of the effect of radical position preference in N400 with greater negativity associated with radicals in preferred position. These findings identify the early ERP components associated with activation of position-specific radical representations in the orthographic lexicon, and reveal the change in the nature of competition from processing at the radical level to the lexical level.

## Introduction

There has been a long-standing and intense interest in alphabetic scripts regarding how positional information of letters in a word is coded (see review of different models in Grainger and Van Heuven, 2003). This is understandable as about 34% of all four-letter words in English and French can form other words by rearranging their letters (Shillcock et al., 2001). Therefore for alphabetic scripts, spatial specification of letter position is vital to correct word recognition and production (Grainger et al., 2006). While the letters are arranged linearly, radicals (also referred to as components or constituents) in a Chinese character are arranged in a two-dimensional square shape. The same question can be raised whether spatial information of components is similarly necessary. That is, are radicals specified for position of occurrence in the orthographic lexicon? The present study investigated the relevance of positional information of orthographic units during the early stages of visual character recognition using event-related potential (ERP) with a primed-lexical decision task.

The Chinese writing system is a non-alphabetic script with words composed of characters that represent morphemes. It has often been characterized as morphosyllabic. Each character occupies a constant square-shaped space that is constructed by combination of stroke patterns. Particular grouping of strokes form radicals that may exist as characters. These radicals may combine to form complex characters (Hoosain, 1991). Over 80% of complex characters contain radicals carrying probabilistic phonetic cues (e.g. 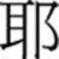, /je4/^1^, is the phonetic radical of 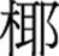,/je4/) or semantic cues (e.g., 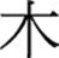 “*wood/plant*” is the semantic radical of 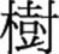 “*tree*”), and their reliability can vary across characters (Chen et al., 1996). It has been reported that up to 10 different spatial arrangement of radicals, or configurations, can be found in complex characters (Fu, 1993), such as horizontal (AB 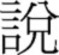, ABC 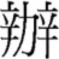), vertical (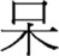), and semi-enclosed configuration (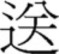).

Studies investigating the properties of radicals have shown that they constitute an important level of representation in the orthographic processing system (e.g., Li and Chen, 1997; Taft et al., 2000), and influence response latencies in lexical decision (e.g., Feldman and Siok, 1997, 1999; Taft and Zhu, 1997), naming (e.g., Taft et al., 1999; Zhou and Marslen-Wilson, 1999; Lee et al., 2005), semantic judgment (e.g., Chen and Weekes, 2004; Chen et al., 2006), and priming tasks (e.g., Ding et al., 2004). They show that character activation is achieved via radical activation in complex characters. For example, Taft and Zhu (1997) found an effect of radical type frequency (number of characters containing that radical irrespective of function) and radical status (real or invented radical) in a lexical decision task.

Growing evidence from electrophysiological studies using ERP has also shown that radicals modulate the N1/N170, frontal P200, and N400 components. Focusing on the phonetic radical, Hsu et al. (2009) found interactive effects of phonetic combinability (neighborhood size of the phonetic radical) and phonological consistency (degree of agreement in pronunciation among orthographic neighbors having the same phonetic radical) at N170 in the occipital region, and main effects of phonetic combinability and phonological consistency at the frontal P200 component. Similar findings were also reported by Lee et al. (2006a,b, 2007) showing that phonetic radical consistency and regularity of the (phonetic) radical was reflected in the frontal P200 component, which Liu et al. (2003) suggested was related to phonological processing. Consistency effects were also found at the late N400 indicating that radicals are involved in later lexical processing via what the authors proposed to be competition of other phonetically similar neighbors (Lee et al., 2006a,b, 2007).

While a number of studies show that the radical acts as a sub-lexical representational unit in the Chinese script, the theoretical question we put forward is to test whether it is necessary to have separate position-specific representations for radicals as argued by Taft and his colleagues in their Multilevel Interactive-Activation framework (Taft and Zhu, 1997; Taft et al., 1999; Taft, 2006). For example, a radical (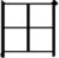) can be located at different positions in a character, such as on the left (e.g., 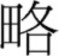), right (e.g., 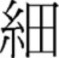), top (e.g., 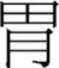), or bottom (e.g., 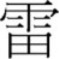; Hoosain, 1991). According to Taft and colleagues, the 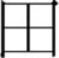 radical in each of the characters above have their own position-specific representation activated (position-specific view), so that the character 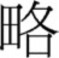 would activate a left-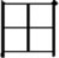 radical, and the character 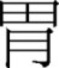 would activate a top-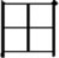 radical. Although all models of Chinese character recognition allow for the representation of radicals, they differ in opinion about how spatial information of these radicals is characterized (e.g., Perfetti et al., 2005; Perfetti and Liu, 2006; Yang et al., 2009).

The claim for separate position-specific representations was based on a study by Taft and Zhu (1997) showing that character decision latency was influenced by radical position frequency (number of characters containing that radical in that position). Specifically, for two characters of equal frequency, the one with high radical position frequency (e.g., the character 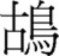 containing 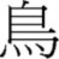 which occurs on the right-hand side of many characters) was easier to be recognized than the one with low radical position frequency (e.g., 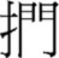 containing 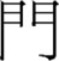 which occurs on the right-hand side of few characters). Taft et al. (1999) also argued that activation of the appropriate position-specific radical unit within a character would lead to lateral inhibition of other inappropriate position-specific radical units as no interference effects were found when recognizing transposable characters using a lexical decision or naming tasks. Thus, characters containing transposable radicals (e.g., 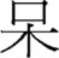 and 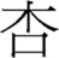) had response latencies and error rates that were comparable to those containing non-transposable radicals (e.g., 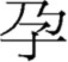). This suggested to them that the same radical occurring at different positions were represented independently. To further support their claim, Ding et al. (2004) show significant priming effects when prime-target characters shared a radical in the same position (e.g., 
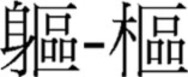
) but not when they shared a radical in different positions (e.g., 
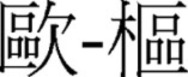
) using a primed-lexical decision task. It was argued that pre-activation of the radical from a similar radical position prime led to faster target lexical decision latencies. However, the priming effect that Ding et al. found is problematic as facilitation in trials with radicals in similar position also shared more visual overlap than when in different positions.

Furthermore, contradictory findings have also been reported, leading some to argue that radicals are not coded for position (position-general view). For instance, the Lexical Constituency Model (Perfetti et al., 2005; Perfetti and Liu, 2006) claims that radicals do not require position information, and postulates instead configuration “slots” to allocate the position-free radicals. This implies that configural information, represented as input units in the model (e.g., left-right or top-bottom), is activated independently from the radicals themselves. Note that Taft’s model also includes position-general radicals that consequently activate position-specific radicals. However, the activation of a complex character is achieved via the activation of its position-specific radicals. This line of reasoning has mainly risen from studies using illusory conjunction and visual search tasks. Tsang and Chen (2009) found using a illusory conjunction task that participants would mistakenly perceive the target character as being one of the two preceding “source” characters when target and source characters have shared radical(s). Importantly, no difference was observed when the shared radical occurred in the same (e.g., 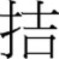 preceded by 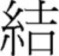 and 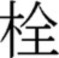) or different (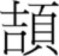 preceded by 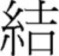 and 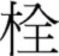) positions across source-target. This finding was taken to argue that radicals were position-general and would activate all characters containing it irrespective of their position. Yet, it is unclear in that study why source characters with radicals in the same position as the target were less error prone than ones in different positions, and a latency difference of approximately 40 ms did not reach statistical significance. Using a visual search paradigm, Yeh and Li (2002) found that target characters took longer to identify when embedded in an array of characters sharing a radical and the same configuration but with the common radical in same or different positions than when they appeared in an array of control (unrelated) characters. Nonetheless, the generalizability of its findings may be challenged with only two target characters manipulated to form eight distractor-target pairs, which were not balanced for character frequency (i.e., 5.18–422.53 per million).

Given the limitations and inconsistencies of previous findings, this study separated the effects of visual overlap resulting from position similarity across prime and target in a primed-lexical decision task, and more significantly took a different conceptual approach to specification of radical position. Spatial position of radicals was explicitly conceived in terms of relative frequency across positions of a radical. As mentioned before, a radical can occupy different positions in characters, with some position encompassing more characters than other positions. For example, the radical 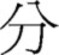 can be found in at least four different positions in characters but it occurs more frequently on the right (76%, e.g., 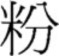) than on the left (9%, e.g., 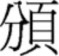), top (2%, e.g., 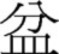), or bottom (4%, e.g., 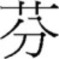), taking into account type-token frequency. Therefore a radical can have a preferred or dominant position (high type-token frequency), while other positions that the radical can also occur in may be considered less preferred or subordinate positions (low type-token frequency). We argue that this conceptualization provides a more controlled test for position-specific radical representations as it relies on the relative frequency of distribution within the radical neighborhood. The contrast between high and low radical position frequency while controlling for character frequency and overall radical frequency in Experiment 2 of Taft and Zhu (1997) can be considered similar to our concept of dominant vs. subordinate positions, although our current design is superior in some important aspects. First, response latencies to characters containing the same radicals were compared as a function of dominant vs. subordinate positions. Unlike in Taft and Zhu where different radicals for high and low radical position frequency were used, without explicitly controlling for factors that may well influence lexical decision latency, including neighborhood size, phonological consistency, and orthographic complexity. Second, our manipulation took into consideration not only type frequency (i.e., number of characters in which a radical appears in a particular position) as in Taft and Zhu, but also token frequency (i.e., the frequency of each of the characters containing the radical in that position), which has been shown to affect the speed of lexical decision (Lee et al., 2005). Finally, the contrast of dominant vs. subordinate positions equally involved the left and right positions or the top and bottom positions of horizontally or vertically structured characters in this study (see details in Materials and Methods), dissimilar to the exclusive focus on the right or bottom position in Taft and Zhu, which may have inadvertently confounded radical position with function. For example, radicals that occur in the right and bottom position are more likely to be phonetic radicals and thus loosely linked to the phonological function. If spatial position information is inherently specified at the radical level, such information should be sensitive to its relative position distribution. Thus, characters with a radical in its dominant position would be recognized faster than those containing a radical in subordinate position, as characters with radicals in preferred (high type-token frequency) locations may require less effort to be activated relative to less preferred (low type-token frequency) locations.

Event-related potential in addition to response latency was collected, as ERP provides excellent temporal resolution and can reveal the unfolding of graphic, phonological, and semantic processes in visual word recognition online (e.g., Perfetti and Tan, 1998; Liu et al., 2003; Hauk et al., 2006; Holcomb and Grainger, 2006). Although previous studies in Chinese character recognition had not investigated ERP components that functionally reflected the processing of position-specific radical representation, ERP components known to reflect semantic and phonetic radical analysis were selected as components of interest, specifically the N1/N170, P200, and N400 components. Using silent naming, Lee and colleagues (Lee et al., 2006a,b, 2007; Hsu et al., 2009) showed that radical processing is associated with the N1/N170, and frontal P200 component, which they suggested reflects activation of radical processing at the visual word form area during the mapping of orthography-to-phonology. Greater N1/N170 negativity was found at electrodes P5/P6, P7/P8, PO5/PO6, and PO7/PO8 for characters that encompassed radicals with high combinability/neighborhoods size (Hsu et al., 2009). They suggest that the N170 component is an index of orthographic detection during the early perceptual categorization stage (see also Bentin et al., 1999), and greater visual experience from highly combinable characters lead to more efficient and specialized processing, thus, showed greater activation at the N170 than low combinability characters.

Lee and colleagues suggested that this early stage of visual word recognition shapes later orthographic-to-phonological conversion of the character’s radical that was reflected in the N170 and more robust at the frontal P200 component (see also Sereno et al., 1998). Sensitivity to the consistency of the (phonetic) radical’s pronunciation within a character showed smaller N170 effects and smaller P200 effects were found at electrodes F3/F4, FC3/FC4, C3/C4, CP3/CP4 Fz, FCZ, FC3, Cz, and CPz for characters that were highly consistent, with the greatest significant difference at the left frontal site F3 (Lee et al., 2007; see also Lee et al., 2006b; Hsu et al., 2009). In a separate task, Liu et al. (2003) showed with a primed pronunciation task that characters sharing similar radicals (graphical similarity) between the prime and target elicited smaller P200 at frontal and central electrodes sites. The N170 and (frontal) P200 components were further found to be sensitive to differences between two types of characters having opposite arrangement of semantic and phonetic radicals (one with semantic radical on the left and phonetic radical on the right vs. one with the opposite alignment; Hsiao et al., 2007). Note though that, this finding could also suggest that character recognition may be sensitive to the positions in which radicals are more likely to occupy as 89.9% of phonetic radicals occur on the right side of characters with semantic radicals on the left of horizontally structured complex characters (Hsiao and Shillcock, 2006).

Radical processing has also been shown to affect the N400 component. Lee et al. (2007) suggests that the N400 reflects a later stage of lexical processing after the P200. Greater N400 component was found for high phonologically consistent radicals at the central region electrodes Fz, FCz, Cz, CPz, and Pz. They argued that due to more homophone characters found in the high consistency condition, greater lexical competition would occur leading to an enhanced N400. In addition, Hsu et al. (2009) found using similar electrodes of interest that highly combinable radicals also elicit a greater N400 component, suggesting that highly combinable radicals increase semantic competition at the N400 (see also Holcomb et al., 2002). Liu et al. (2003) also identified that characters preceded by visually similar primes showed smaller N400 amplitudes at the central region during a semantic relatedness judgment task.

In light of previous ERP findings, it is assumed that position information is encoded early (Taft, 2006), and will modulate the early stages of character recognition in which visual-perceptual analysis proceeds to the orthographic stage as reflected in the N1/N170 and P1 visual components, but may also influence the frontal P200 and N400 (Liu et al., 2003; Lee et al., 2007; Hsu et al., 2009). It is predicted that characters with similar radical position to their primes will show a more negative P1, N1/N170, and reduced N400 due to prior exposure from the prime, while the position effect of dominance may be reflected in the later components, including the N170 and N400 components.

## Materials and Methods

### Participants

Twenty-five native Cantonese speakers aged 18–23 (*M* = 20.8, SD = 1.7; female: 14) participated in the study. Three participants were excluded in the ERP analysis due to excessive movements or loss of over 40% of useable trials (*M* = 20.95, SD = 1.64; female = 11). All were assessed to be right-handed (Oldfield, 1971), had normal or corrected-to-normal vision, and no prior history of learning difficulties, reading difficulties or head injury. All had completed their secondary education in local mainstream schools and not lived outside of Hong Kong for more than 2 years.

### Materials

Seventeen radicals of interest were selected that could appear in dominant and subordinate positions, respectively, in at least four relatively low frequency characters (token frequency less than 100 in a million) using the Hong Kong Corpus of Chinese NewsPaper (HKCCNP; Leung and Lau, 2010) database. The degree of dominance was calculated by dividing the sum frequency of characters that shared the same radical in each possible position by the total frequency of all characters sharing the radical irrespective of position (i.e., dominance  = position token frequency/radical token frequency × 100). Radicals that can occur in more than one possible position in characters and appear in one particular position over 60% of the time were classified as radicals having a dominant position of occurrence. The positions in which these radicals appear less than 35% of the time were classified as subordinate. For each of the 17 radicals, four characters containing it were selected, two serving as target and two as prime. The prime and target characters would be paired in such a way that the target radical occurred in either its dominant or subordinate position in the target character, preceded by the prime character containing the radical in either the same or different position. For example, the radical 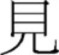 appears on the right-hand side of a character, such as 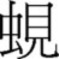, 86% of the time (hence its dominant position), and at the bottom of a character, such as 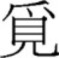, 11% (hence its subordinate position). When 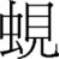 and 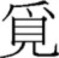 were selected as target characters for the dominant and subordinate position conditions, respectively, they would each be matched with two prime characters with 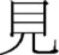 in either the right side 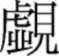 or the bottom 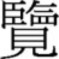. This is illustrated in Table 1.

**Table 1:** **Examples of pairs of prime (left) and target (right) characters with mean dominance, frequency (per million), and stroke number in each experimental condition**.

	Radical dominance	
	Dominant	Subordinate
**Position Distribution (SE)**	75.88% (2.91)	9.25% (2.25)
	[60–92%]	[0.1–31%]
**RADICAL POSITION**		
**Same**	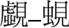	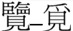
Frequency	14.58–7.92	25.04–7.97
Stroke	12.06–12.12	12.35–11.53
**Different**	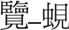	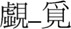
Frequency	25.04–7.92	14.58–7.97
Stroke	12.35–12.12	12.06–11.53

Of the 17 target radicals, the dominant and subordinate positions appear in characters of the same configuration in 12 cases. The dominant position of eight radicals occurs on the left, seven on the right, one each in the top and the bottom. Six of the radicals serve as a semantic radical in a character, seven as a phonetic radical, and four as either.

All the target characters and prime characters were matched in character frequency [*t*(33) = 1.35, *p* = 0.19] and visual complexity in terms of stroke number [*t*(33) = 1.14, *p* = 0.26]. To represent the variety of character configurations found in Chinese, we included both characters in horizontal and vertical configurations. Phonological and semantic similarities between primes and targets were avoided as much as possible.

The same number of pseudo characters, created by combining the target radicals with other radicals in their legal positions was used as fillers. These were stroke matched to the target characters. All the prime-target pairs were pseudo-randomized for each participant to avoid successive exposure to the same prime or target. There were 34 primes and 68 targets.

All stimuli were presented as digitized images measuring 3 mm × 33 mm (125 × 125 pixels) and presented in yellow MingLiu font on a black background. The forward and backward mask consisted of a 125 × 125 pixels matrix with half of the pixels randomly colored in yellow and the other half in black.

### Procedure

The participants took part in a primed-lexical decision task where they were asked to judge whether the target character was a real or pseudo character as quickly and accurately as possible. Each trial began with a fixation cross (500 ms), sequentially followed by a blank page that randomly varied in duration for 500–700 ms (*M* = 601 ms), a forward mask (100 ms), the prime character (96 ms), a backward mask (16 ms), and finally the target character, which remained on the screen until the participant made a response. Once a response was made, a blank screen (500 ms) appeared, followed by an “eye blink” cue (500 ms) and another blank screen for a random duration between 800 and 1000 ms (*M* = 897 ms). The eye blink cue was used to reduce blinking artifacts occurring in the critical time windows of interest. Fifteen practice trials were given to each participant, and a total of 204 experimental trials were divided evenly into four blocks randomized across participants.

Participants were seated in front of a LCD monitor (60 frames/s) at a distance of approximately 100 cm, in an electrically and acoustically shielded room. Before the experiment began, the participants were instructed to minimize their movements and eye blinks to reduce artifactual electroencephalography (EEG) signals. The E-prime 2.0 (Psychological Software Inc.) program was used to present the stimuli, collect behavioral reaction time, and accuracy data. Across all participants, the response hand for lexical decision was counter-balanced.

#### EEG recording and pre-processing

Electroencephalography/ERP data recorded from 128 Ag/AgCl electrodes (NSL QuikCap, Neuromedical Supplies, Sterling, USA) was digitized online at 1 kHz and amplified with a band pass of 0.05–200 Hz using SynAmps2^®^ (Neuroscan, Inc., El Paso, TX, USA) amplifiers. All electrodes were referenced to a common vertex electrode between electrodes 63 (equivalent to Cz) and 64 (CPz), and ground (GND) was positioned anterior to electrode 60 (Fz). Horizontal eye movement was measured using a pair of bipolar electrodes placed approximately 1 cm lateral to the left and right external canthi (HEOG). Eye blinks and vertical eye movements were monitored using two bipolar electrodes placed on the supra- and infraorbital ridges of the left eye (VEOG). Electrode impedance was maintained below 5 KΩ as much as possible.

In the off-line analysis, channels with bad recording were first removed, ranging from none to three electrodes across participants. The remaining data was subsequently filtered using a zero phase low-pass filter of 30 Hz (12 dB/octave slopes). Channels affected by eye blink were corrected mathematically using individually modeled eye blinks computed from at least 100 eye blink artifacts for each participant based on the ocular artifact reduction procedure implemented in Scan 4.5 (Neuroscan, Inc). Epochs of real character trials (−400 pre-stimulus onset to 1000 ms post-stimulus onset intervals) were then selected and baseline corrected using a 100 ms pre-stimulus interval before the presentation of the forward mask (pre-target stimulus interval of −312–212 ms). Incorrect trials and trials with voltage exceeding ±60 μV or affected by muscle movements were automatically rejected, with equivalent amount of trials being excluded across the experimental conditions (Dominant-same = 22.51%; Dominant-different = 22.76%; Subordinate-same = 24.55%; Subordinate-different = 23.79%). The remaining data was then re-referenced to the average activity across all channels and used to compute grand average waveforms for each condition per participant.

### Data analysis

A within-participant and between-item two-way analysis of variance (ANOVA) was used to analyze the behavioral reaction time and percentage of error data. Target radical dominance (dominant vs. subordinate) and radical position similarity (same vs. different) between the prime and target served as the independent measures. In both analyses, *post hoc* multiple comparisons were adjusted using the Bonferroni correction.

**Figure 1:**
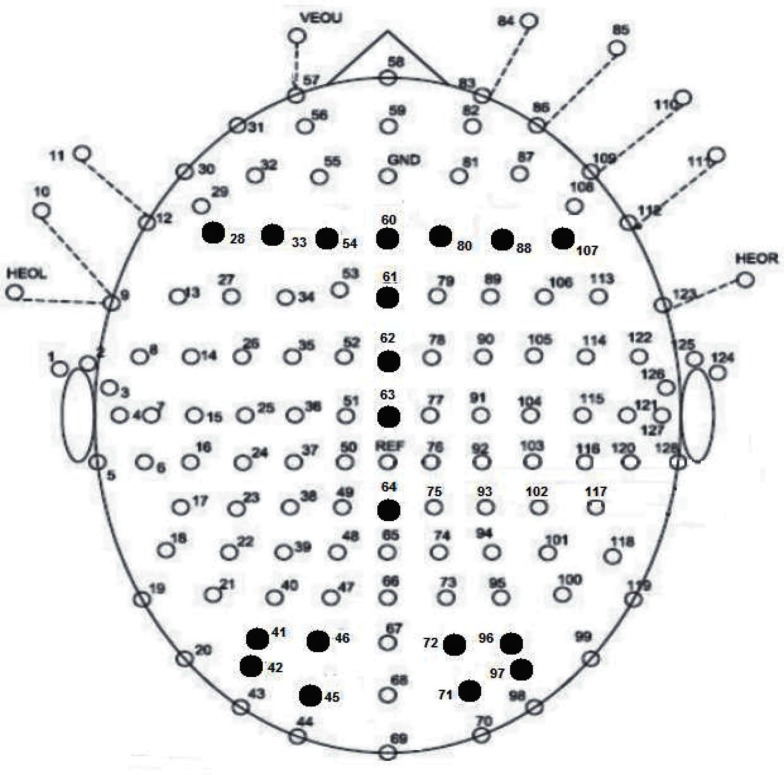
**Topographic plot of electrode array (128 NSL Quikcap) with electrodes filled in black used in statistical analysis**.

For the analysis of ERP data, within-participant three-way ANOVA was conducted at the N400 component, with the inclusion of electrode location as the third independent variable. At the occipital (N1, P1, and N170) and frontal component (P200) analyses, within-participants four-way ANOVA was implemented with hemisphere (left vs. right) included. The mean amplitudes at *a priori* selected electrodes served as the dependent variable. Bonferroni adjustment was used to correct the significance threshold for *post hoc* comparisons, and the Greenhouse–Geisser (ε) correction was applied when the assumption of sphericity of variance was violated. The electrodes and components were selected *a priori* based on electrode locations found in previous radical analysis studies and were expected to display maximal amplitudes correlated with visual word form processing at the occipital N1, P1, and N170 component analyses (Holcomb and Grainger, 2006, 2009; Lee et al., 2007; Hsu et al., 2009), phonological processing at the frontal electrodes for the P200 component analysis (Rugg, 1984; Sereno et al., 1998; Lee et al., 2007; Hsu et al., 2009), and semantic processing reflected along the midline electrodes for the N400 component analysis (Liu et al., 2003; Lee et al., 2006a,b, 2007), respectively. The time windows for the components of interest were selected based on the Mean Global Field Power (MGFP) of all trials. Thus, the mean amplitudes at the N1, P1, and N170 components, were computed between 50–100, 120–180, and 225–325 ms, respectively, at the left parietal-occipital electrodes 41, 42 (PO5), 45 and 46 (PO3), and right parietal-occipital electrodes 96, 97 (PO6), 71 and 72 (PO4; see Figure 1 for electrode array of selected channels). At the frontal P200 component, the mean amplitude was computed between 225 and 325 ms and the electrodes of interest were at the left frontal electrodes 28 (F5), 33 (F3), and 54 (F1), and right frontal electrodes 107 (F6), 88 (F4), and 80 (F2). As the N400 component is maximal at the central region, a time window of 300–450 ms was chose for five electrodes, 60 (Fz), 61 (FCz), 62, 63 (Cz), and 64 (CPz) along the midline.

## Results

### Behavioral results

The pseudo character filler trials and five trials with response latencies exceeding 3000 ms (<0.01) were discarded. Trials exceeding ±2.5 SD from the mean of each participant were also excluded from the analysis (1.77%). The mean response latencies for each condition and accuracy are shown in Table 2.

**Table 2:** **Mean RT and accuracy of each experimental condition**.

Dominance	Prime position	Reaction time		Accuracy	
		*M* (ms)	SE	*M* (%)	SE
Dominant	Same	676	23.28	88.1	0.02
Dominant	Different	702	22.28	90.4	0.02
Subordinate	Same	685	30.63	89.4	0.02

With the remaining data, a two-way ANOVA with target radical dominance (dominant or subordinate) and radical position (same or different) was conducted. Only a main effect of radical position was found, *F*(1, 24) = 5.37, *p* < 0.05, $\eta_{p}^{2}$ = 0.18, where participants were significantly faster to recognize the target character when the prime and target shared a radical in the same position (*M* = 680.62 ms, SE = 26.07) than in a different position (*M* = 696.03 ms, SE = 23.03). No main effect of radical dominance, *F*(1, 24) = 0.01, *p* = 0.928, $\eta_{p}^{2}$ = 0, or interaction, *F*(1, 24) = 1.09, *p* = 0.306, $\eta_{p}^{2}$ = 0.04, was observed, suggesting that characters with radicals in their dominant or subordinate position did not affect the speed of character recognition.

Error analysis showed no significant effects indicating that accuracy was not affected by the dominance of the radical’s position in the target character, the similarity in radical position between prime and target, or their interaction (all *p*’s > 0.05).

### ERP results

**Figure 2:**
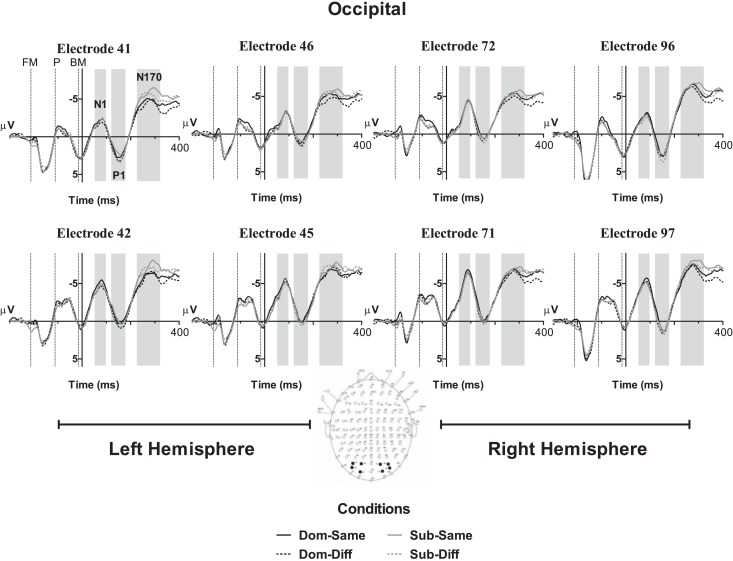
**Grand averaged ERP waveforms showing effects of radical dominance and radical position at N1, P1, and N170 components located at the parietal-occipital electrodes and shaded in gray**. Dom-Same, dominant radical character with prime radical in same position; Dom-Diff, dominant radical character with prime radical in different position; Sub-Same, subordinate radical character with prime radical in same position; Sub-Diff, subordinate radical character with prime radical in different position. Dotted vertical lines indicate the onset of the forward mask (FM), prime (P), and backward mask (BM).

**Figure 3:**
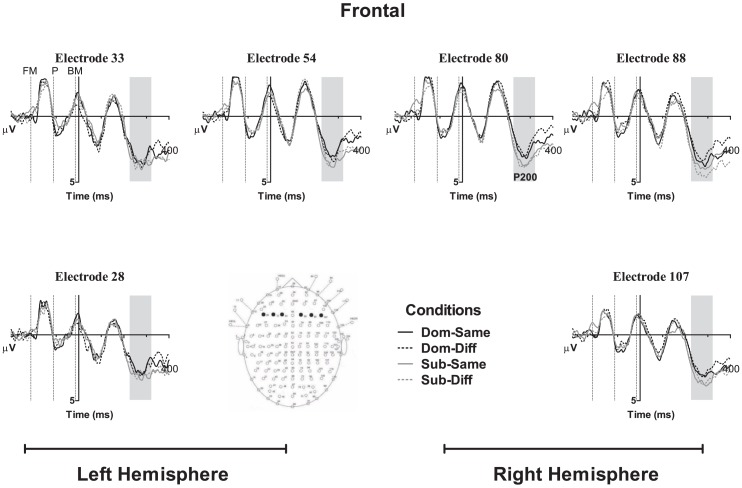
**Grand averaged ERP waveforms showing effects of radical dominance and radical position at the frontal P200 component shaded in gray**. Dom-Same, dominant radical character with prime radical in same position; Dom-Diff, dominant radical character with prime radical in different position; Sub-Same, subordinate radical character with prime radical in same position; Sub-Diff, subordinate radical character with prime radical in different position. Dotted vertical lines indicate the onset of the forward mask (FM), prime (P), and backward mask (BM).

**Figure 4:**
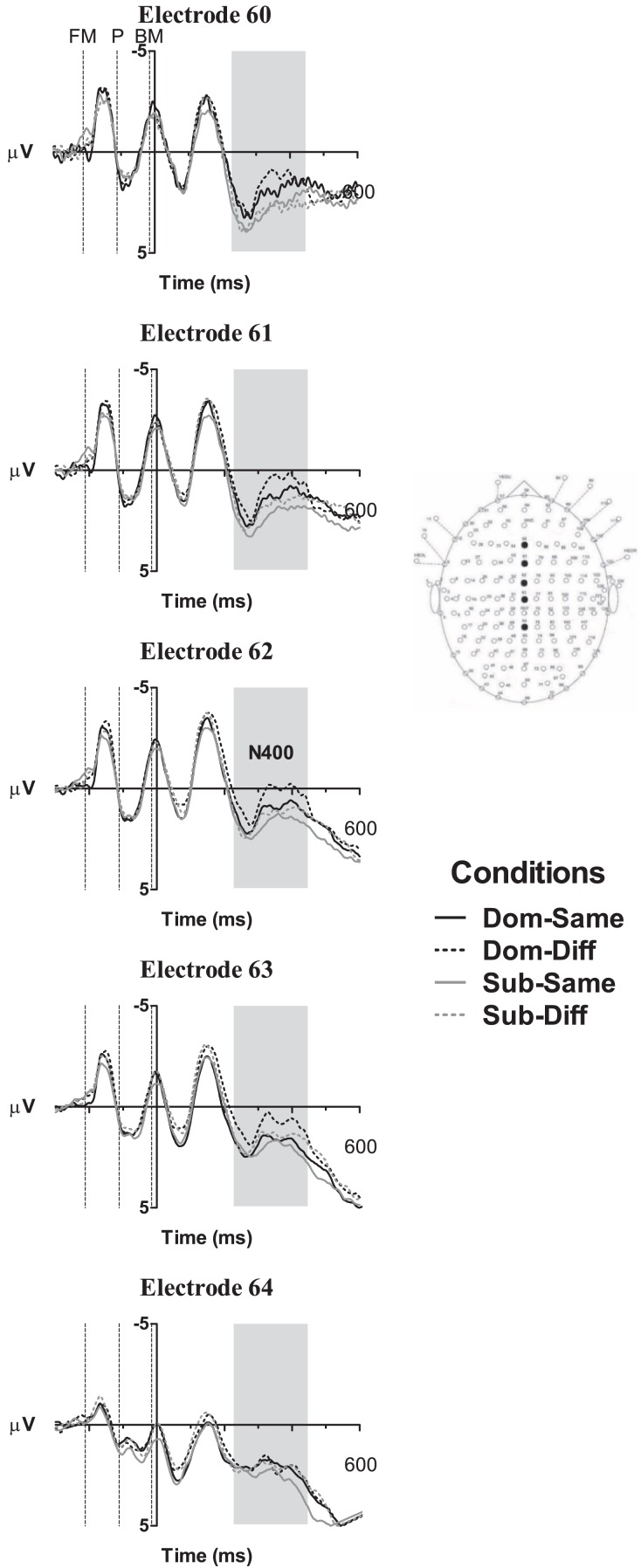
**Grand averaged ERP waveforms showing effects of radical dominance and radical position at the N400 component located at the centro-parietal midline electrodes and shaded in gray**. Dom-Same, dominant radical character with prime radical in same position; Dom-Diff, dominant radical character with prime radical in different position; Sub-Same, subordinate radical character with prime radical in same position; Sub-Diff, subordinate radical character with prime radical in different position. Dotted vertical lines indicate the onset of the forward mask (FM), prime (P), and backward mask (BM).

**Figure 5:**
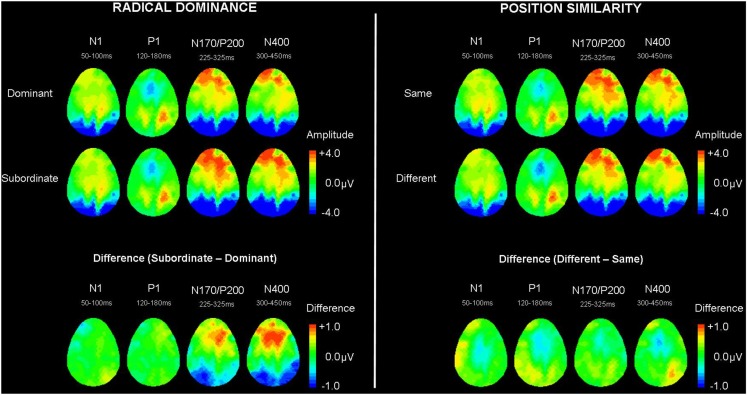
**Grand averaged topographic plots showing scalp amplitude activity and amplitude differences for the effects of radical dominance (left) and radical position similarity (right)**. Dom, dominant radical character; Sub, subordinate radical character; Same, same radical position of prime radical to character; Diff, different radical position of prime radical to character.

Based on the MGFP, the ERP morphology started its first negative deflection with a maximal peak in the occipital regions at 83 ms from stimulus onset followed by a positive deflection at 151 ms and negative deflection at 279 ms. The frontal electrodes showed a similar pattern to the N170 in the occipital region but with its polarity reversed; hence, occipital N170-frontal P200. A later central negativity peaking at 383 ms and positivity at 585 ms were also observed. Figures 2–4 show the grand average waveforms for the effects of radical dominance and radical position at various components in the frontal, centro-parietal, and occipito-parietal electrodes. Topographic plots showing scalp distribution and difference amplitude for radical dominance and position similarity effects are shown in Figure 5.

#### N1 (50–100 ms)

No significant effects were found in the four-way repeated measures ANOVA at the first occipital component, all *p*’s > 0.05.

#### Occipital P1 (120–180 ms)

At the next occipital component, the four-way repeated measures ANOVA showed a main effect of radical position, *F*(1, 21) = 4.62, *p* < 0.05, $\eta_{p}^{2}$ = 0.18, indicating that target characters preceded by primes with radical in a different position elicited a more positive amplitude (*M* = 0.83 μV, SE = 0.63) than in the same position (*M* = 0.31 μV, SE = 0.63), see Figure 2. No main effect of radical dominance was observed, *F*(1, 21) = 1.28, *p* = 0.27, $\eta_{p}^{2}$ = 0.06, nor interactions with hemisphere or electrode, all *F*’s < 2.37, *p*’s > 0.10.

#### Occipital N170 (225–325 ms)

At the later occipital N170 component in Figure 2, the effect of radical dominance was found *F*(1, 21) = 6.21, *p* < 0.05, $\eta_{p}^{2}$ = 0.24, such that characters with radicals in the subordinate position (*M* = −6.07 μV, SE = 0.97) elicited a more negative going potential than targets with radicals in their dominant position (*M* = −5.48 μV, SE = 0.90). However, the effect of radical position was not significant, *F*(1, 21) = 0.23, *p* = 0.63, $\eta_{p}^{2}$ = 0.01, or any of the interactions, all *F*’s < 3.57, *p*’s > 0.07.

#### Frontal P200 (225–325 ms)

The frontal P200 component illustrated in Figure 3 also shows effects of radical dominance similar to the occipital N170, *F*(1, 21) = 9.19, *p* < 0.005, $\eta_{p}^{2}$ = 0.30, whereby characters with subordinate radicals (*M* = 3.16 μV, SE = 0.60) elicited greater positivity than characters with dominant radicals (*M* = 2.49 μV, SE = 0.55). The effect of radical position was again, not significant, *F*(1, 21) = 0.04, *p* = 0.80, $\eta_{p}^{2}$ < 0.01. Moreover, no interactions were observed, all *F*’s < 1.95, p’s > 0.17.

#### N400 (300–450 ms)

The three-way ANOVA with electrodes along the midline revealed main effects of radical dominance, *F*(1, 21) = 21.00, *p* < 0.005, $\eta_{p}^{2}$ < 0.36, showing that characters with a dominant radical (*M* = 1.27 μV, SE = 0.54) elicited a more negative going wave than characters with a subordinate radical (*M* = 2.00 μV, SE = 0.53). Radical position similarity was also significant, *F*(1, 21) = 5.37, *p* < 0.05, $\eta_{p}^{2}$ = 0.20, and characters with radicals in different positions to their primes (*M* = 1.36 μV, SE = 0.48) showed greater negativity than characters with radicals in the same position (*M* = 1.89 μV, SE = 0.58).

No interactions were shown to be significant, including the radical dominance-by-position interaction, *F*(1, 21) = 0.12, *p* = 0.74, $\eta_{p}^{2}$ = 0.01, as well as the by electrode interactions *F*_Elect-by-position_ (1.72, 36.20) = 1.20, *p* = 0.31, $\eta_{p}^{2}$ = 0.05, ε = 0.43; *F*_Elect-by-dominance_ (1.94, 40.79) = 2.57, *p* = 0.09, $\eta_{p}^{2}$ = 0.10, ε = 0.49.

To summarize the main ERP findings, the occipital P1 component showed a main effect of radical position similarity whereby primes and targets sharing a radical in the same position exhibited a less positive P1 than cases with a common radical in different positions. At the later occipital N170/frontal P200, greater negativity and positivity, respectively, were found for characters with target radicals in their subordinate position compared to characters with target radicals in the dominant position. However, while the pattern of the effect of radical position similarity remained principally the same for the N400 component where radicals appearing in different positions in the prime and target revealed greater negativity than those with radicals in the same position, the pattern of radical dominance effects changed. More specifically, characters containing radicals in dominant positions elicited a larger N400 than characters with the same radicals in subordinate positions, particularly at the central-frontal sites.

## Discussion

The aims of this study were to assess Taft and colleagues’ claim for independent representation of position-sensitive radicals and to identify ERP components that may reflect position-specific radical processing and the stage(s) at which the associated effects take place. Unlike previous studies, this investigation separated the effect of visual overlap from that of position specification in radicals by manipulating whether prime-target pairs shared similar radical positions or whether the target’s radical differed in relative position frequency factorially. While the behavioral results only showed a position similarity effect, the ERP findings revealed a more complex contribution/relationship of radical processing during the stages of lexical processing. Radical dominance effects were found at the occipital N170-frontal P200 and N400, in addition to the observations of visual similarity effects at the early occipital P1 and later N400 components. Neural sensitivity to radical dominance supports the view that position-specific radicals are activated early, and that the spatial relationship among orthographic units within a character can impact on character recognition. The following discussion considers the position similarity effect reflected in response latency, and examines each of the significant ERP components and attempts to integrate the temporal dynamics of radical processing into Taft’s model of character processing (Taft et al., 1999; Taft, 2006).

Based on the behavioral effects, when a target character shared a radical in similar position to its prime, participants were faster to recognize the target due to pre-activation of the relevant position-specific constituent radical in the prime facilitating the recognition of the target character. Previously, this has been taken as evidence for position-specific information of radical representation (Taft and Zhu, 1997; Ding et al., 2004), but such an interpretation may be problematic. First, the ERP findings confirmed that the facilitation was primarily driven by visual overlap between the prime and target radical rather than from independent representations of position-sensitive radicals *per se*. This was because radical position effects were found in early components known to reflect visual analysis at the P150 component in the occipital regions (Grainger and Holcomb, 2009). Less activation was needed when primes and targets shared a radical in the same position compared to different positions, particularly in the left hemisphere. Moreover, although the radical position effects found at the N400 could be indicative of position-specific radical representational processing as radicals in different positions required more effort for integration as they elicited greater N400, one may nevertheless argue that such an observation would suggest that the independent radical representations only influence lexical processing.

Contrasting the effects of radical position and radical dominance in relation to the time course of Chinese character recognition, the findings suggest that visual overlap/similarity is initially processed before access to radical representation as radical position effect precedes the radical dominance effect. The effects of radical dominance provide stronger support for independent representation of position-specific radicals, considering that it is unaffected by the degree of visual overlap between prime and target. Greater negativity at occipital N170 and positivity at frontal P200 for characters with radicals in subordinate position may be taken to reflect increased processing effort to activate the less frequently encountered subordinate radical representation. On the other hand, dominant radical representations are frequently activated because they are connected to more characters containing the radicals in the same positions, and thereby have a lower activation threshold. As such, characters with radicals in dominant positions would require less effort to process, at least initially. This pattern of less activation for characters with radicals in dominant position, however, changes at the later N400 component when character recognition proceeds from radical level facilitation to lexical level competition. Note that radical position effects however, continue to show a smaller N400 component for target characters sharing a similar radical position to their primes. At the N400 component, characters with dominant position radicals elicited greater negativity compared with subordinate position. We argue that radical processing at the N400 reflects competition at the lexical level (see also Lee et al., 2007) as dominant radical position characters naturally have more neighbors that are simultaneously activated, and thus may require greater effort and/or lateral inhibition to suppress irrelevant neighboring competitors to select the appropriate lexical entry. Characters with subordinate position radicals, on the other hand, co-activate a smaller set of neighbors and would therefore involve less conflict resolution. An issue, however, arises that the N400 is generally considered to be sensitive to lexico-semantic features and assumed to represent post-lexical semantic integration or access to semantic representations in the long term memory (e.g., Kutas and Hillyard, 1980, 1984; Nobre and McCarthy, 1994; Barber and Kutas, 2007). However, the N400 time window of 300–450 ms in this study is earlier than the typical N400 associated with post-lexical semantic integration; thus, the authors argue that the N400 here may capture the earlier phase of the N400 (see Grainger and Holcomb, 2009, for a similar interpretation of ERP masked repetition priming effects). All models of Chinese character recognition take the view that radicals serve as perceptual input only (Taft et al., 1999; Perfetti et al., 2005; Perfetti and Liu, 2006; Taft, 2006), whether the N400 component continues to reflect lexical competition at the word form level or interference at the semantic level from non-target neighbors requires further investigation. We are, however, inclined to argue that the interference reflected in the earlier N400 in our findings occurs at the word form level as equal numbers of semantic and phonetic radicals served as target radicals, and all prime-target pairs were semantically unrelated. Another pertinent observation is that the main effect at the P200 and N400 show similar topographic difference distribution suggesting that the P200 effect argued to be at the sub-lexical level could be related to the later N400. However, we favor the view that the N400 effect is a lexical event associated with the more conventional interpretation of lexical level competition. Nonetheless, what is clear is that position specified radicals play a crucial role in character recognition in Chinese, and affect this process in a complex manner that behavioral experiments alone cannot reveal. Specifically, changes from facilitation to competition of the position-specific radicals from the radical level to the lexical level may offset each other, and result in a null effect of radical dominance in RT.

The presence of radical dominance effects at the occipital N170 could be seen as reflecting neighborhood size effects (Chen and Weekes, 2004; Chen et al., 2006; Hauk et al., 2009; Hsu et al., 2009) or a neighborhood-by-position interaction (Grainger et al., 2006). The observation of greater processing effort for characters with subordinate radicals is consistent with findings of neighborhood size effects reported from behavioral, functional imaging, and ERP studies in the form of facilitative effects for words with larger neighborhoods (Hsiao et al., 2007; Hsu et al., 2009, 2011; Li et al., 2010). Hsiao et al.’s (2007) study showing position effects at occipital N170 and frontal P200 components can also be described as a type of neighborhood-by-position bias effects between two types of characters having opposite distribution of semantic and phonetic radicals of left-right configuration characters. The dominant radical positions in this study can be analogous to their SP condition where the phonetic radical occurs on the right side of character and left for semantic radical (S and P denote the semantic and phonetic radical, respectively). The less preferred or subordinate position has the opposite alignment (PS). In this case, greater negativity at the occipital N170 or positivity at the frontal P200 for the less preferred positions of radicals is similar to our findings. However, our study differs significantly from Hsiao et al. as it takes a more parsimonious approach of not conflating radical position with functional specificity (see also Zhou and Marslen-Wilson, 1999). As radicals assuming particular functions are more likely to be located in particular positions, the relationship between radical position and functional units still requires delineating. If indeed the radical dominance effects can be conceptualized to be associated with the neighborhood size effect, we suggest that the N170 could reflect a lexico-orthographic component in which the representation of the word form neighborhood is processed. Based on Grainger and Holcomb (2010), the time course of the N170 component in the current study, corresponding to their N250, suggests that activation from orthographic units (e.g., radicals) to words (e.g., character) may take place in this time window with its neural generator located at the left fusiform gyrus region associated with the visual word from area (VWFA). In relating the N170 to N250 in Grainger and Holcomb, it is notable that the time course of our N170 (as well as P1) peaks at a similar time to Grainger and Holcomb (i.e., P150 and N250) which use a similar masked priming design. This may account for the delay of our N170 compared to previous ERP studies with Chinese radicals which typically used covert naming (e.g., Lee et al., 2007) or semantic judgment tasks (e.g., Liu et al., 2003) without a mask.

Finally, the early N1 component observed in this study may be a consequence of the prime’s N1 component overlapping with the target character, and reflect a delayed N1 activity of the prime. This may explain the null effects and more crucially its early peak latency of the N1 at 75 ms (prime + backward mask = 112 ms). The typical visual N1 component peaks at around 120–150 ms.

To integrate the present results within the context of Taft’s (2006) interactive-activation model in terms of the temporal dynamics of radical processing, we propose that position-general radicals are initially activated at the occipital P1 component at approximately 150 ms, and subsequently spread activation to their position-coded radicals. Around 280 ms in the occipital N170/frontal P200 component, character representations containing position-specific radicals are activated and lexical selection of the word form (or semantics) occurs at approximately 380 ms at the N400, with greater negativity reflecting greater lexical competition.

In conclusion, our results support the role of position-specific radicals in orthographic processing as proposed by Taft (2006) and Taft et al. (1999). This study importantly separates facilitative effects due to visual overlap from position information of radicals and proposes a temporal framework for radical processing. Distinct position-specific representations are conceptualized in terms of dominance of radical position via relative type-token frequency, and we demonstrated that independent position-specific radicals are activated within the first 250 ms of character recognition. Such early access is revealed via modulation of the occipital N170/frontal P200 component, followed by the retrieval of lexico-orthographic or lexico-semantic information at the N400. Our evidence for position-specific radical representations, therefore, highlights the over simplication of the Lexical Constituency Model (Perfetti et al., 2005) as an account of Chinese character processing. While character configuration may be relevant, it alone may not be adequate to access word form representation.

## Conflict of Interest Statement

The authors declare that the research was conducted in the absence of any commercial or financial relationships that could be construed as a potential conflict of interest.

## Footnotes

^1^Phonetic transcriptions of Chinese characters provided in this paper are given in jyutping, a Romanization system developed by the Linguistic Society of Hong Kong. The number in the transcription represents the tone.

## References

[B1] Barber H. A. Kutas M. (2007). Interplay between computational models and cognitive electrophysiology in visual word recognition. Brain Res. Rev. 53, 98–123 10.1016/j.brainresrev.2006.07.002 16905196

[B2] Bentin S. Mouchetant-Rostaing Y. Giard M. H. Echallier J. F. Pernier J. (1999). ERP manifestations of processing printed words at different psycholinguistic levels: time course and scalp distribution. J. Cogn. Neurosci. 11, 235–260 10.1162/089892999563373 10402254

[B3] Chen M. J. Weekes B. S. (2004). Effects of semantic radicals on Chinese character categorisation and character decision. Chin. J. Psychol. 46, 181–196

[B4] Chen M. J. Weekes B. S. Peng D. L. Lei Q. (2006). “Effects of semantic radical consistency and combinability on Chinese character processing,” in The Handbook of East Asian Psycholinguistics, 1st Edn, Vol. 1: Chinese, eds Li P. Bates E. Tan L. H. Tzeng O. J. L. (Cambridge: Cambridge University Press), 175–186

[B5] Chen Y. P. Allport D. A. Marshall J. C. (1996). What are the functional orthographic units in Chinese word recognition: the stroke or the stroke pattern? Q. J. Exp. Psychol. A 49, 1024–1043 10.1080/713755668

[B6] Ding G. Peng D. Taft M. (2004). The nature of the mental representation of radicals in Chinese: a priming study. J. Exp. Psychol. Learn. Mem. Cogn. 30, 530–539 10.1037/0278-7393.30.2.530 14979822

[B7] Feldman L. B. Siok W. W. T. (1997). The role of component function in visual recognition of Chinese character. J. Exp. Psychol. Learn. Mem. Cogn. 23, 776–781 10.1037/0278-7393.23.3.776 9165709

[B8] Feldman L. B. Siok W. W. T. (1999). Semantic radicals contribute to the visual identification of Chinese characters. J. Mem. Lang. 40, 559–576 10.1006/jmla.1998.2629

[B9] Fu Y.-H. (1993). “The structure and construction of Chinese characters,” in Computational Analysis of Modern Chinese Characters, ed. Chen Y. (Shanghai: Shanghai Educational Press), 108–169 [in Chinese].

[B10] Grainger J. Granier J.-P, Farioli, F. van Assche E. van Heuven W. J. B. (2006). Letter position information and printed word perception: the relative-position priming constraint. J. Exp. Psychol. Hum. Percept. Perform. 32, 865–884 10.1037/0096-1523.32.4.865 16846285

[B11] Grainger J. Holcomb P. J. (2009). Watching the word go by: on the time-course of component processes in visual word recognition. Lang. Linguist. Compass 3, 128–156 10.1111/j.1749-818X.2008.00121.x 19750025 PMC2740997

[B12] Grainger J. Holcomb P. J. (2010). “Neural constraints on a functional architecture for word recognition,” in The Neural Basis of Reading, eds Cornelissen P. Hansen P. Kringlebach M. Pugh K. (Oxford: Oxford University Press), 3–32

[B13] Grainger J. Van Heuven W. J. B. (2003). “Modeling letter position coding in printed word perception,” in The Mental Lexicon, ed. Bonin P. (New York: Nova Science Publishers), 1–24

[B14] Hauk O. Davis M. H. Ford M. Pulvermüller F. Marslen-Wilson W. D. (2006). The time course of visual word recognition as revealed by linear regression analysis of ERP data. Neuroimage 30, 1383–1400 10.1016/j.neuroimage.2005.11.048 16460964

[B15] Hauk O. Pulvermüller F. Ford M. Marslen-Wilson W. D. Davis M. H. (2009). Can I have a quick word? Early electrophysiological manifestations of psycholinguistic processes revealed by event-related regression analysis of the EEG. Biol. Psychol. 80, 64–74 10.1016/j.biopsycho.2008.04.015 18565639

[B16] Holcomb P. J. Grainger J. (2006). On the time course of visual word recognition: an event-related potential investigation using masked repetition priming. J. Cogn. Neurosci. 18, 1631–1643 10.1162/jocn.2006.18.10.1631 17014368 PMC1808538

[B17] Holcomb P. J. Grainger J. (2009). ERP effects of short interval masked associative and repetition priming. J. Neurolinguistics 22, 301–312 10.1016/j.jneuroling.2008.06.004 20161320 PMC2678731

[B18] Holcomb P. J. Grainger J. O’Rourke T. (2002). An electrophysiological study of the effects of orthographic neighborhood size on printed word perception. J. Cogn. Neurosci. 14, 938–950 10.1162/089892902760191153 12191460

[B19] Hoosain R. (1991). Psycholinguistic Implications for Linguistic Relativity: A Case Study of Chinese. Hillsdale, NJ: Lawrence Erlbaum Associates

[B20] Hsiao J. H. Shillcock R. (2006). Analysis of a Chinese phonetic compound database: implications for orthographic processing. J. Psycholinguist. Res. 35, 405–426 10.1007/s10936-006-9022-y 16897357

[B21] Hsiao J. H. Shillcock R. Lee C. (2007). Neural correlates of foveal splitting in reading: evidence from an ERP study of Chinese character recognition. Neuropsychologia 45, 1280–1292 10.1016/j.neuropsychologia.2006.10.001 17098263 PMC1876781

[B22] Hsu C. H. Lee C. Y. Marantz A. (2011). Effects of visual complexity and sublexical information in the occipitotemporal cortex in the reading of Chinese phonograms: a single-trial analysis with MEG. Brain Lang. 117, 1–11 10.1016/j.bandl.2010.10.002 21111475

[B23] Hsu C. H. Tsai J. L. Lee C. Y. Tzeng O. J. L. (2009). Orthographic combinability and phonological consistency effects in reading Chinese phonograms: an event-related potential study. Brain Lang. 108, 56–66 10.1016/j.bandl.2008.09.002 18951624

[B24] Kutas M. Hillyard S. A. (1980). Reading senseless sentences: brain potentials reflect semantic incongruity. Science 207, 203–208 10.1126/science.7350657 7350657

[B25] Kutas M. Hillyard S. A. (1984). Brain potentials during reading reflect word expectancy and semantic association. Nature 307, 161–163 10.1038/307161a0 6690995

[B26] Lee C.-Y. Tsai J.-L. Chan W.-H. Hsu C.-H. Hung D. L. Tzeng O. J. L. (2007). Temporal dynamics of the consistency effect in reading Chinese: an event-related potentials study. Neuroreport 18, 147–151 10.1097/WNR.0b013e3282f22998 17301680

[B27] Lee C.-Y. Tsai J.-L. Chiu Y.-C. Tzeng O. J. L. Hung D. L. (2006a). The early extraction of sublexical phonology in reading Chinese pseudocharacters: an event-related potentials study. Lang. Linguist. 7, 619–635

[B28] Lee C.-Y. Tsai J.-L. Huang H.-W. Hung D. L. Tzeng O. J. L. (2006b). The temporal signatures of semantic and phonological activations for Chinese sublexical processing: an event-related potential study. Brain Res. 1121, 150–159 10.1016/j.brainres.2006.08.117 17011529

[B29] Lee C.-Y. Tsai J.-L. Su E. C.-I. Tzeng O. J. L. Hung D. L. (2005). Consistency, regularity and frequency effects in naming Chinese characters. Lang. Linguist. 6, 75–107

[B30] Leung M. T. Lau K. Y. D. (2010). Hong Kong Corpus of Chinese NewsPaper. The University of Hong Kong [Unpublished database].

[B31] Li H. Chen H.-C. (1997). “Processing of radicals in Chinese character recognition,” in The Cognitive Processing of Chinese and Related Asian Languages, ed. Chen H.-C. (Hong Kong: Chinese University Press), 141–160

[B32] Li Q. L. Bi H. Y. Zhang J. X. (2010). Neural correlates of the orthographic neighborhood size effect in Chinese. Eur. J. Neurosci. 32, 866–872 10.1111/j.1460-9568.2010.07327.x 20626458

[B33] Liu Y. Perfetti C. A. Hart L. (2003). ERP evidence for the time course of graphic, phonological, and semantic information in Chinese meaning and pronunciation decisions. J. Exp. Psychol. Learn. Mem. Cogn. 29, 1231–1247 10.1037/0278-7393.29.6.1231 14622057

[B34] Nobre A. C. McCarthy G. (1994). Language-related ERPs: scalp distributions and modulations by word type and semantic priming. J. Cogn. Neurosci. 6, 233–255 10.1162/jocn.1994.6.3.233 23964974

[B35] Oldfield R. C. (1971). The assessment and analysis of handedness: the Edinburgh inventory. Neuropsychologia 9, 97–113 10.1016/0028-3932(71)90067-4 5146491

[B36] Perfetti C. A. Liu Y. (2006). “Reading Chinese characters: orthography, phonology, meaning, and the Lexical Constituency Model,” in The Handbook of East Asian Psycholinguistics, 1st Edn, Vol. 1: Chinese, eds Li P. Bates E. Tan L. H. Tzeng O. J. L. (Cambridge: Cambridge University Press), 225–236

[B37] Perfetti C. A. Liu Y. Tan L. H. (2005). The lexical constituency model: some implications of research on Chinese for general theories of reading. Psychol. Bull. 112, 43–59 10.1037/0033-295X.112.1.43 15631587

[B38] Perfetti C. A. Tan L. H. (1998). The time course of graphic, phonological, and semantic activation in Chinese character identification. J. Exp. Psychol. Learn. Mem. Cogn. 21, 24–33 10.1037/0278-7393.29.6.1231 14622057

[B39] Rugg M. D. (1984). Event-related potentials and the phonological processing of words and non-words. Brain Lang. 23, 225–240 10.1016/0093-934X(84)90065-8 6518354

[B40] Sereno S. C. Rayner K. Posner M. I. (1998). Establishing a time-line of word recognition: evidence from eye movements and event-related potentials. Neuroreport 9, 2195–2200 10.1097/00001756-199807130-00009 9694199

[B41] Shillcock R. Ellison T. M. Monoghan P. (2001). Eye-fixation behavior, lexical storage, and visual word recognition in a split processing model. Psychol. Rev. 107, 824–851 10.1037/0033-295X.107.4.824 11089408

[B42] Taft M. (2006). “Processing of characters by native Chinese readers,” in Handbook of East Asian Psycholinguistics, 1st Edn, Vol. 1: Chinese, eds Li P. Tan L. H. Bates E. Tzeng O. J. L. (Cambridge: Cambridge University Press), 237–249

[B43] Taft M. Zhu X. (1997). Submorphemic processing in reading Chinese. J. Exp. Psychol. Learn. Mem. Cogn. 23, 761–775 10.1037/0278-7393.23.3.761

[B44] Taft M. Zhu X. Ding G. (2000). The relationship between character and radical representation in Chinese. Xin Li Xue Bao 32(Suppl.), 3–12

[B45] Taft M. Zhu X. Peng D. (1999). Positional specificity of radicals in Chinese character recognition. J. Mem. Lang. 40, 498–519 10.1006/jmla.1998.2625

[B46] Tsang Y.-K. Chen H.-C. (2009). Do position-general radicals have a role of play in processing Chinese characters? Lang. Cogn. Process. 24, 947–966 10.1080/01690960802154615

[B47] Yang J. McCandliss B. Shu H. Zevin J. (2009). Simulating language-specific, and language-general effects in a statistical learning model of Chinese reading. J. Mem. Lang. 61, 238–257 10.1016/j.jml.2009.05.001 20161189 PMC2728242

[B48] Yeh S.-L. Li J. L. (2002). Role of structure and component in judgments of visual similarity of Chinese characters. J. Exp. Psychol. Hum. Percept. Perform. 28, 933–947 10.1037/0096-1523.28.4.933 12190259

[B49] Zhou X. Marslen-Wilson W. (1999). The nature of sublexical processing in reading Chinese characters. J. Exp. Psychol. Learn. Mem. Cogn. 25, 819–837 10.1037/0278-7393.25.4.819

